# Evaluation of Satisfaction with the Built Environment of University Buildings under the Epidemic and Its Impact on Student Anxiety

**DOI:** 10.3390/ijerph20054183

**Published:** 2023-02-26

**Authors:** Qiang Wen, Haiqiang Liu, Jinyuan Chen, Huiyao Ye, Zeyu Pan

**Affiliations:** 1 School of Civil Engineering and Architecture, Zhejiang Sci-Tech University, Hangzhou 310018, China; 2 Department of Architecture, Zhejiang University, Hangzhou 310018, China

**Keywords:** academic building, built environment, satisfaction evaluation, anxiety, natural exposure, physical conditions

## Abstract

Anxiety on college campuses has increased due to the COVID-19 epidemic’s profound effects on society. Much research has been conducted on how the built environment influences mental health; however, little has been undertaken on how it affects student mental health in the context of the epidemic from the architectural scale perspective of academic buildings. Based on online survey data, this study develops multiple linear regression and binary logistic regression models to investigate students’ satisfaction ratings of the academic buildings’ physical environments during the epidemic and how these satisfaction ratings affect students’ anxiety tendencies. According to the study’s findings regarding the natural exposure perspective, students who perceived the academic building’s poor semi-open space view (*p* = 0.004, OR = 3.22) as unsatisfactory factors were more likely to show anxiety tendencies. In terms of the physical conditions, students who were dissatisfied with the noise level in the classroom (*p* = 0.038, OR = 0.616) and the summer heat in semi-open spaces (*p* = 0.031, OR = 2.38) were more likely to exhibit anxiety tendencies. Additionally, even after controlling for confusing distractions, the general satisfaction rating of the academic building’s physical environments (*p* = 0.047, OR = 0.572) was still able to significantly and negatively affect students’ anxiety tendencies. The study’s findings can be used in the architectural and environmental planning of academic buildings focusing on mental health.

## 1. Introduction

COVID-19 outbreaks are linked to poor mental health outcomes, including anxiety and depression symptoms [1]. In 2020, over 41 million students were enrolled in higher educational institutions [2], and college students were more likely to experience anxiety and depression, with 15% of Chinese college students suffering anxiety symptoms [3]. As the outbreak was effectively contained, students’ mental health improved slightly [4], but the closed management of most campuses also contributed to mental health issues among college students.

The built environment is the physical environment constructed for human life and activities, which has an impact on people’s physical health [5,6,7] as well as their psychological health (e.g., anxiety and depression) [8,9,10,11]. In recent years, extensive research has been conducted on the relationship between the built environment and mental health [12,13,14,15], including residential floor levels [16], green spaces [17,18], and exposure to indoor air pollutants [19]. The built environment can impact people’s mental health by influencing their connection to nature, personal control, and indoor air quality [20]. On the other hand, some studies do not conclusively demonstrate a connection between noise, building form, or a green environment and mental health [21].

Crowding, poor indoor air circulation, and ambient air pollution are risk factors for COVID-19 transmission in the built environment [22]. Higher ambient levels of delicate particulate matter and nitrogen oxides are associated with an increased risk of COVID-19 morbidity, severity, and mortality [22]. On the other hand, the frequency of use of green space and green window views in the home was related to higher levels of self-worth, life satisfaction, and subjective well-being during the COVID-19 epidemic, as well as lower levels of depression, anxiety, and loneliness [23]. During an isolation lockdown, staying in touch with nature (blue-green space) lowered the risk of developing depressive and anxiety symptoms [24] and was found to mitigate the adverse effects of social isolation on mental health [25,26].

There are several related studies on the campus’ built environment’s effect on students’ mental health during an epidemic. Students’ perceptions of the built environment, attitudes, and walking patterns on campus during an epidemic may impact their physical and mental well-being [27]. In an isolated shelter area, preferred interior colors, indoor plants, artwork, and high-quality green and sky views can all lower the risk of anxiety and depression [28]. The campus environment during the epidemic had the greatest psychological recovery effect for blue spaces, followed by green spaces, sports fields, while it was least pronounced in gray spaces [29].

According to existing research, aside from the stress induced by academic pressure, the epidemic’s development exacerbated students’ anxiety [3], and the campus’s built-up environment also impacted students’ mental health and anxiety [28,29]. The majority of research on the relationship between the built environment and mental health has been completed on large-scale built environments [12,21] or small-scale residential buildings [9,16]; fewer studies have been conducted at the architectural scale of academic buildings, particularly in the context of epidemics. Furthermore, current research focuses on the independent effects of specific factors on mental health, such as green space [17,24], air pollution [19], and traffic noise [30]. While these factors are usually spatially correlated as built environment features, separately evaluating them would ignore the potential confounding effects between them.

The closed campus management, which forced students to spend more time in the academic building in the context of the epidemic, caused students to have harmful psychological emotions. The academic building’s physical environment can have a more substantial effect on students’ mental health. However, the built environment is rarely created with design aspects deliberately intended to enhance mental health [31]. At the scale of academic buildings, the built environment has three dimensions: the interior environment, the semi-open space environment, and the external perimeter environment, which includes both physical conditions and natural exposure. This article aims to examine how satisfied students are with each aspect of the academic building’s constructed environment in the context of the epidemic, as well as how these ratings affect students’ tendency for anxiety. In order to prevent the interference of confounding factors, a multi-factor holistic model was created for the study. With the aim of reducing student anxiety and enhancing mental health through improvements to the built environment of academic buildings, the study’s findings will aid in relevant decision-making and serve as a reference for the design and renovation of the architecture and environment of academic buildings.

The rest of the document is organized as follows. Section 2 covers the relevant theory and the formulation of hypotheses; Section 3 covers the study design, variable factors, and statistical methods; Section 4 presents the statistical results; Section 5 provides an analytical discussion of the results, including the study’s limitations and future work; and Section 6 summarizes the key findings and conclusions.

## 2. Related Studies

### 2.1. Natural Exposure and Mental Health

Studies have shown that exposure to nature has a positive effect on improving physical health, mental health [32], and cognitive functioning [33]; that reduced exposure has a negative impact on mental health outcomes [20]; and that individuals who live in areas with a lack of green space and who are physically unhealthy are more likely to experience mental health issues [34]. During the COVID-19 epidemic, frequent use of green space and the presence of a view from a home’s windows were linked to higher levels of self-worth, life satisfaction, and subjective well-being, as well as lower levels of sadness, anxiety, and loneliness [23]. Staying in touch with nature (blue-green space) can lessen the risk of experiencing symptoms of sadness and anxiety [24] and can buffer or lessen the negative impacts of social isolation on mental health [25,26]. According to studies conducted from a campus perspective, campus greenery significantly enhances students’ physical health. It lowers physical stress [35], and exposure to natural settings can have a positive effect on academic performance [36,37,38]. Good window views can positively affect students’ mental health and mood, as well as their ability to concentrate [39,40]. In conclusion, natural exposure to the built environment has an enormously beneficial impact on mental health. We tested the hypothesis that students unsatisfied with the natural exposure component in the built environment would be more prone to anxiety.

### 2.2. Physical Conditions and Mental Health

From the perspective of ventilation and mental health, studies have shown that air pollution caused by poor ventilation is associated with adverse mental health outcomes [20,41,42,43] and that indoor air pollutants cause adverse health effects, ranging from respiratory diseases [44] to cognitive effects [45,46,47]. Fine airborne particles (PM2.5) formed by poor ventilation have been linked to symptoms of depression and anxiety [45], which play an essential role in depression and psychotic disorders [48,49]. Low ventilation rates increase indoor carbon dioxide concentrations, a potential health risk [50]. In addition, air pollution can deter participation in outdoor activities, which can affect mental health [51,52]. During the outbreak, higher levels of ambient delicate particulate matter and nitrogen oxides were connected to an elevated risk of COVID-19 morbidity, severity, and fatality [22].

According to research on the thermal environment and mental health associations, cold temperatures reduce negative mental health outcomes while hot temperatures increase them [53]. High temperatures have been associated with depression, anxiety, mood disorders, aggression [54], a decrease in positive mood, an increase in negative mood, and fatigue [55]. They may also exacerbate psychiatric conditions and impact mental health [56], as well as increase the risk of suicide and hospitalization for mental illness [57]. In addition, studies have found a relationship between the thermal environment of the classroom and student learning efficiency [58].

In terms of the relationship between noise and mental health, several studies have shown that noise has a detrimental effect on mental health [30,59,60], that annoyance brought on by noise exposure is positively associated with anxiety and depression [59], that urban and traffic noise is related to adverse mental health outcomes, and that neighborhood noise disturbance increases the likelihood of having poor mental health [30].

According to research, daylight and illumination are related to reduced weariness, relief from melancholy, reduced depressive symptoms, and many other health advantages [61], with inadequate lighting increasing the likelihood of developing depression by 60% [31]. Quality lighting has been linked to decreased stress, anxiety, and improved mood [62]. Furthermore, studies show that lighting that produces visual comfort increases health, well-being, and satisfaction, improving learning and visual performance [63].

In summary, physical conditions in the built environment have a significant impact on mental health. Many studies have shown that air pollution, heat and noise exposure, and poor illumination negatively impact mental health. Therefore, we tested the following hypothesis: students dissatisfied with physical conditions will show a greater tendency to be anxious.

## 3. Materials and Methods

### 3.1. Study Design

This study first compiled the built environment factors of the academic building based on existing literature, then designed questionnaires and collected data based on the study’s purpose, modeled the research data, conducted the statistical analysis, and discussed it with existing studies to reach its conclusions. This paper investigated student satisfaction ratings for each aspect of the academic building’s built environment in the context of the epidemic and the relationship between these satisfaction ratings and student anxiety tendencies. Based on the earlier analysis and the model’s streamlining through variable screening, 16 built environment factors were used as independent variables. General satisfaction and anxiety tendencies were used as dependent variables to create multiple linear regression and binary logistic models, respectively, to cut out the interference of confounding factors and investigate the independent influence.

On the “Questionnaire Star” platform, a self-administered computerized survey was used to collect the data. During the COVID-19 epidemic in April 2022, college campuses routinely established a strictly controlled system of access to the campus. They prohibited all students from being on campus in order to control the spread of the new coronavirus outbreak. We recruited college students from 139 universities in various provinces to participate in the questionnaire study to ensure the data collection’s representativeness and value. After the screening, 241 valid research questionnaires were found out of 279 that had been gathered. The reliability tests showed that the scale questions were consistent and that the study data were highly reliable (Cronbach’s Alpha = 0.928). There is some association between the topics, and the validity of the study data is good, according to the findings of the validity test in the factor analysis (KMO value = 0.923, estimated chi-square = 1605.185, *p* < 0.05).

### 3.2. Built Environment Factors

Table 1 depicts the constructed environment at the scale of the academic building analyzed in this work, which includes both natural exposure and physical conditions, as well as three dimensions of the inside, semi-open area, and outside perimeter of the teaching structure. Watching nature, being with nature, and seeking physical connection with nature are three components of nature exposure that have a positive impact on mental health [64], so natural exposure at the scale of a teaching building had five components: the indoor landscape view of classrooms, the number of semi-open spaces, semi-open space landscape view, surrounding landscape and leisure facilities, and surrounding green vegetation. An approximate 50-m radius was used to outline the academic building’s surrounding boundary. After field research, we found that students often closed the curtains to shelter themselves from direct sunlight when using the classroom and that the classroom landscape view was only accessible from the window seats, the study of the classroom internal landscape view was discarded. Additionally, the physical conditions included four dimensions: ventilation, thermal environment, classroom noise, and classroom lighting, with ventilation including three aspects, namely indoor ventilation, semi-open space ventilation, and ventilation around the teaching building. The thermal environment likewise included three aspects: the indoor thermal environment, the semi-open space thermal environment, and the outdoor thermal environment around the teaching building.

### 3.3. Questionnaire Composition and Variables

The questionnaire included five parts, as shown in Table 2, the first of which asked about personal traits and background. Four independent factors were included in this section: gender, grade, climate zone, and inner or outer corridor. According to the “What is your school?” survey, the variable “climate zone” was discovered. The data was divided into four categories: “severe cold regions”, “cold regions”, “hot summer and cold winter regions”, and “hot summer and warm winter regions.” The second part concerned students’ overall satisfaction with the built environment and whether they tended to be anxious. Anxiety symptoms were measured using the Generalized Anxiety Inventory (GAD-7) [65]. The third, fourth, and fifth sections, with a total of 14 independent variables, focused on students’ satisfaction evaluations of each aspect of the built environment of the academic building, including the indoor environment, outdoor environment, and semi-open space environment. The indoor environment consisted of four items: classroom ventilation, lighting, noise, and thermal comfort, while the outdoor environment also consisted of four items: landscape and leisure facilities, green vegetation, wind environment, and thermal environment around the academic building. The semi-open space environment consisted of six items: quantity, wind environment, landscape view, winter cold, summer heat, and semi-open space satisfaction. A Likert scale was used for indoor and outdoor environmental factors and semi-open space satisfaction, indicating students’ satisfaction with each factor of the built environment of the academic building on a 5-point scale from “very dissatisfied” to “very satisfied.” The sub-elements of the semi-open space environment of the academic building are dichotomous variables, expressing “whether they are unsatisfactory factors”.

### 3.4. Statistical Methods

The data were statistically analyzed using SPSS 26.0 software. First, descriptive statistics were run, which included demographic factors, background characteristics, students’ general satisfaction with the built environment, whether they were anxious, and satisfaction with each built environment factor. All variables were then subjected to a correlation analysis matrix and co-linearity test with a *t*-test and a chi-square test to exclude those variables with a large autocorrelation and no significant effect on the dependent variable. A tolerance greater than 0.1 or a variance inflation factor less than 5 indicates no covariance. A correlation coefficient below 0.8 indicates no strong correlation between the variables. The model was simplified to obtain the 17 variables used in the subsequent modeling.

In order to investigate which factors of the built environment significantly affect students’ evaluation of overall satisfaction with the built environment, a multivariate linear regression model was used with “general satisfaction” as the dependent variable and 16 other variables as independent variables. A binary logistic regression model was developed to investigate the built environment factors that may influence college students’ anxiety tendencies, with “anxiety tendencies” as the dependent variable and the satisfaction rating of each built environment factor of the academic building as the independent variable.

## 4. Results

### 4.1. Descriptive Statistics

The samples were primarily concentrated in hot summer and cold winter regions (57.7%) and hot summer and warm winter regions (27.4%), with the remainder located in severe cold regions (2.9%) and cold regions (12%). There were significantly more inner corridors (76.6%) than outer corridors (22.4%) in the academic building. Juniors (21.2%) and seniors (44.8%) constituted most of the sample grades. The Generalized Anxiety Scale (GAD-7) scores range from 0 to 21 on a scale of normal (0 to 4), mild anxiety (5 to 9), moderate anxiety (10 to 14), and severe anxiety (15 to 21). With a cut-off score of 5, respondents were divided into two groups: those without anxiety tendencies (GAD-7 score <5) and those with anxiety tendencies (GAD-7 score ≥ 5) [65]. By the statistics, as shown in Table 3, there were significantly more students without anxiety tendencies (77.6% of the whole sample) than students with anxiety tendencies (22.4% of the entire sample). Male students had an anxiety rate of 22.7%, while female students had an anxiety rate of 22.1%.

**Figure 1:**
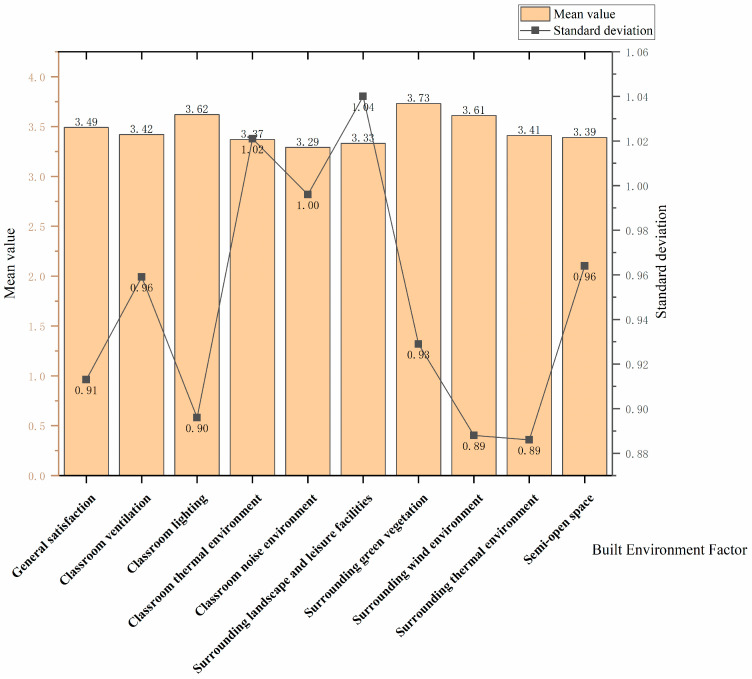
Description of built environment factor satisfaction.

The general satisfaction rating (M = 3.49, SD = 0.91) of students with the built environment of the academic building was ordinary. As shown in Figure 1, among all built environment factors, students were least satisfied with the classroom noise environment (M = 3.29) and most satisfied with the surrounding green vegetation (M = 3.73). Students’ satisfaction with the surrounding landscape facilities (SD = 1.04) had the greatest variability in their opinion ratings, and their satisfaction with the surrounding thermal environment (SD = 0.89) had the least variability in their opinion ratings.

As shown in Table 4, college students were more satisfied with the outdoor environment around the academic building (M = 3.52), the internal environment of the classroom was average (M = 3.425), and the semi-open space environment of the academic building was rated the lowest (M = 3.39). In the indoor classroom environment, students had the highest satisfaction rating for lighting (M = 3.62, SD = 0.90) and the lowest satisfaction rating for noise (M = 3.29, SD = 1.00). During the epidemic, students’ range of activities was restricted, and their sensitivity to noise increased. Noises from the surrounding traffic environment, adjacent classrooms, and corridors may be the main sources of noise disturbance. Additionally, the indoor thermal environment (M = 3.37, SD = 1.02) and the ventilation environment (M = 3.42, SD = 0.96) received average satisfaction ratings. In the outdoor environment surrounding the academic building, students rated the highest level of satisfaction with the green vegetation environment (M = 3.73, SD = 0.93) and the lowest with the landscape and leisure facilities (M = 3.33, SD = 1.04), indicating that students have a higher demand for the surrounding landscape and leisure facilities. Concerning physical conditions, the wind environment surrounding the academic building was assessed as good (M = 3.61, SD = 0.89), while satisfaction with the thermal environment was rated as average (M = 3.41, SD = 0.89).

**Figure 2:**
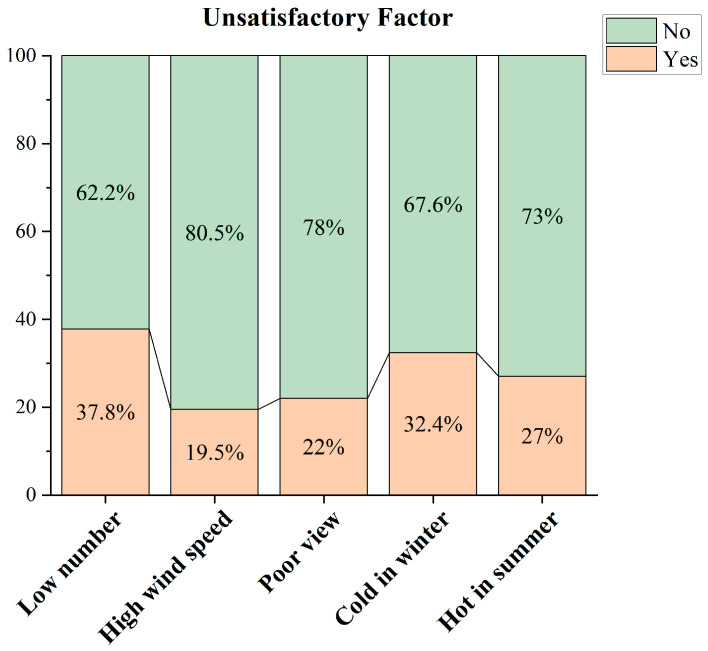
Evaluation of unsatisfactory factors of semi-open space.

The factor “low number” (37.8%) was the most frequently chosen in the questionnaire survey on respondents’ dissatisfaction with the semi-open space environment in the academic building, followed by “cold in winter” (32.4%), “hot in summer” (27.0%), “poor view of the landscape” (22.0%), and “high wind speed” (19.5%), as shown in Figure 2.

### 4.2. Variable Screening and Model Refinement

#### 4.2.1. Co-Linearity Test

A co-linearity test is required to bring the dependent and independent variables of the logistic regression directly into the linear regression model to obtain the tolerance and variance inflation factors and avoid the covariance problem between variables, which affects the correct estimation of the regression model. Table 5 displays the results; the variance inflation factors are all less than 5, with the largest value being “Surrounding wind environment” (3.702), and the model has no multicollinearity issues.

#### 4.2.2. Correlation Check

In order to eliminate the covariance problem, a Pearson correlation matrix analysis was undertaken on the independent variables of all scale questions to screen out the significantly more correlated independent variables. Table 6 shows that the correlation coefficients between each independent variable were less than 0.8, with the “surrounding wind environment” and “surrounding green vegetation” having the strongest correlation (r = 0.765). Additionally, it can also be noted that the connection between each built environment aspect and general satisfaction with the built environment of the academic building ranged from 0.532 to 0.618, with the “surrounding wind environment” having the lowest correlation (r = 0.532).

#### 4.2.3. One-Factor Test

In order to remove independent variables that were not substantially different in the binary logistic regression model, a chi-square cross-tabulation test was performed between the categorical independent variables and the dependent variable (anxiety tendencies). As demonstrated in Table 7, “semi-open space cold in winter” (*p* = 0.863) and “high wind speed in semi-open space” (*p* = 0.855) were not significantly different from “anxiety tendencies” among the semi-open space dissatisfaction variables. In terms of the demographic background variables, “gender” (*p* = 0.913), “grade” (*p* = 0.36), “climate zone” (*p* = 0.102), and “inner or outside corridor” (*p* = 0.684) did not differ significantly for “anxiety tendencies” and were therefore included as control variables and not further analyzed. On the other hand, the continuous independent variable of the scale questions was analyzed using an independent samples *t*-test with “anxiety tendencies”. According to Table 8, “surrounding wind environment” (*t* = 1.073, *p* = 0.284) was the least significant independent variable with the dependent variable.

A chi-squared cross-tabulation was conducted between the categorical independent factors and the dependent variable (general satisfaction) of the multiple linear regression model. As shown in Table 9, among the demographic background variables, “climate zone” significantly differed from “general satisfaction” (*p* = 0.011). In contrast, “gender”, “grade”, and “inner or outside corridor” did not (*p* = 0.533, *p* = 0.781, and *p* = 0.684, respectively). “high wind speed in semi-open space” (*p* = 0.813), “semi-open space cold in winter” (*p* = 0.268), and “semi-open space hot in summer” (*p* = 0.142) were not significantly different from “general satisfaction” among the semi-open space dissatisfaction factors.

#### 4.2.4. Summary of Independent Variable Screening

To simplify the model with a small sample of cases, the co-linearity test, correlation test, and one-way analyses (chi-square test, *t*-test) were used to examine the relationships between all variables. Furthermore, independent variables with higher covariance and those that did not significantly differ from the dependent variable might be eliminated. Combined with the comprehensive analysis from the professional point of view, the excluded independent variables were “surrounding wind environment”, “high wind speed in semi-open space”, and “semi-open space cold in winter”. Table 10 shows the remaining 16 variables and models developed, with demographic background variables (gender, grade, climate zone, inner or outer corridor) included as control variables not to be analyzed in the study.

### 4.3. Multiple Linear Regression Analysis

A multivariate linear regression model was developed with “general satisfaction” as the dependent variable and 16 other variables as independent variables. The stepwise method was used in building the multiple linear regression model. The regression equation was statistically significant (F = 42.265, *p* < 0.05), and the coefficient of determination of the model (R^2^ = 0.593) suggested that the eight significant independent variables explained 59.3% of the general satisfaction with the built environment. The Durbin-Watson test value of 1.906 indicates that the samples are independent.

Table 11 shows the coefficient table for the multiple linear regression model. The results show that the factors that significantly and positively affected the general satisfaction of the built environment were “classroom ventilation” (beta = 0.194, *t* = 3.252, *p* = 0.001), “classroom thermal environment” (beta = 0.176, *t* = 2.810, *p* = 0.005), “classroom noise environment” (beta = 0.138, *t* = 2.365, *p* = 0.019), “surrounding landscape and leisure facilities” (beta = 0.290, *t* = 5.307, *p* < 0.001), and “surrounding thermal environment” (beta = 0.150, *t* = 2.397, *p* = 0.017). The overall satisfaction rating of the built environment was not significantly impacted by students’ satisfaction with “classroom lighting”, “poor view of semi-open space”, “semi-open space hot in summer”, “low number of semi-open spaces”, or “surrounding green vegetation”.

According to the size of the standardized coefficient, “surrounding landscape and leisure facilities” (beta = 0.290), “classroom ventilation” (beta = 0.194), “classroom thermal environment” (beta = 0.176), “surrounding thermal environment” (beta = 0.150), and “classroom noise environment” (beta = 0.138) played the most significant role in influencing general satisfaction with the built environment. In the context of the epidemic, the factors influencing students’ overall satisfaction evaluation of the built environment are primarily the surrounding landscape and leisure facilities, classroom ventilation, classroom thermal and acoustic settings, and so on.

### 4.4. Binary Logistic Regression Analysis

In order to evaluate the influence of each built environment satisfaction factor on students’ anxiety tendencies, a binary logistic regression model was designed with “anxiety tendencies” as the dependent variable and 16 additional built environment factors as independent variables. Since in the previous study, as shown in Section 4.2, we screened and streamlined the independent variables, we used the entry method in building the binary logistic regression model to comprehensively study each independent variable’s effect on the dependent variable. As shown in Table 12, the Omnibus tests revealed that the model was statistically significant (*p* < 0.05, F = 63.66). The Hosmer-Lemeshow test revealed that the model was well-fitted (*p* = 0.17 > 0.05), and the −2 log-likelihood value was 184.14, which quantitatively evaluated the model fit. The model successfully classified 79.9% of the observed samples, 31.4% of which were predicted to have anxiety tendencies and 93.1% of which were predicted to have no anxiety tendencies.

The absolute magnitude of the regression coefficients, as shown in Table 13, indicates the degree of influence of each influencing factor on students’ anxiety tendencies; the positive and negative signs indicate the direction of influence; and the OR values indicate the probability of occurrence of anxiety tendencies relative to the reference group. Statistical results showed the independent variables that significantly influenced students’ anxiety tendencies were: “poor view of semi-open space” (B = 1.169, *p* = 0.004, OR = 3.220), “semi-open space hot in summer” (B = 0.867, *p* = 0.031, Exp(B) = 2.380), “classroom noise environment” (B = −0.485, *p* = 0.038, OR = 0.616), and “general satisfaction” (B = −0.559, *p* = 0.047, OR = 0.572). On the other hand, “classroom ventilation”, “classroom lighting”, “classroom thermal environment”, “surrounding landscape and leisure facilities”, “surrounding green vegetation”, “surrounding thermal environment”, “semi-open space satisfaction”, and “the low number of semi-open spaces” had no significant effect on the development of students’ anxiety tendencies.

**Figure 3:**
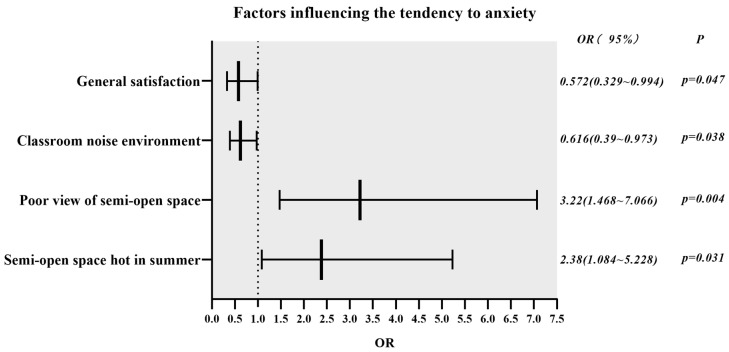
Factors affecting the tendency to anxiety.

The poor view of semi-open space had the most significant effect on the probability of anxiety occurrence among students, followed by the semi-open space hot in summer, both of which positively affected the probability of anxiety tendencies among students, as shown in Figure 3. The general satisfaction with the built environment of the academic building and the satisfaction with the classroom noise environment negatively affected the probability of student anxiety. Among dissatisfaction factors with semi-open space, “poor view of semi-open space” could significantly and positively affect students’ anxiety tendencies (OR = 3.220, *p* = 0.004). Students who perceived a poor view of semi-open space as a dissatisfactory factor had a 2.22-fold increased risk of anxiety. “Semi-open space hot in summer” influenced students’ anxiety significantly and positively (OR = 2.380, *p* = 0.031). Students who perceived summer heat in semi-open spaces as a dissatisfaction factor were 1.38 times more likely to have anxiety.

Even after controlling for the confounding effects of other variables, “general satisfaction with the built environment” showed an independently significant, negative influence on students’ anxiety (OR = 0.572, *p* = 0.047). With each level of improvement from “very dissatisfied” to “very satisfied” about the students’ overall satisfaction with the built environment, their anxiety tendencies declined by 0.43 times. The “classroom noise environment” could significantly and negatively affect the probability of students’ anxiety tendencies (OR = 0.616, *p* = 0.038). As students’ satisfaction with the classroom noise environment increased by one level from “very dissatisfied” to “very satisfied”, the probability of having anxiety decreased by 0.38 times.

## 5. Discussion

### 5.1. Main Findings

This study discovered that the semi-open space design and the classroom noise environment are the most critical characteristics that can significantly influence students’ anxiety tendencies in the context of the epidemic and at the scale of the academic building. Semi-open space landscape views and summer heat are all characteristics of semi-open space that can significantly affect student anxiety. The evaluation of the semi-open space landscape view, in particular, played the most important role in determining whether students were anxious or not. The semi-open space view variable represents the method of natural contact. The summer hot in semi-open spaces reflects the physical conditions of semi-open spaces. Other natural exposure factors, such as “surrounding green vegetation”, “surrounding landscape and leisure facilities”, and “low number of semi-open spaces”, did not significantly affect students’ anxiety tendencies. Regarding other physical conditions, only “classroom noise environment” and “semi-open space hot in summer” significantly influenced the probability of students’ anxiety tendencies. “Classroom ventilation”, “classroom lighting”, and “classroom thermal environment”; “surrounding thermal environment”, “surrounding wind environment”; “high wind speed in semi-open space”, “semi-open space cold in winter”; all did not affect students’ tendency to anxiety. It is noteworthy that general satisfaction with the built environment, excluding the confounding effect of other variables, can still significantly affect the probability of students’ anxiety tendencies.

Regarding physical conditions, the factors that can significantly influence students’ general satisfaction evaluation of the built environment include “classroom ventilation”, “classroom thermal environment”, “classroom noise environment”, and “surrounding thermal environment”. “Classroom lighting”, “surrounding wind environment”, “surrounding thermal environment”, and “high wind speed in semi-open space” did not significantly affect students’ general satisfaction evaluation of the built environment. In terms of natural contact, the landscape and leisure facilities around the academic building significantly affected the overall satisfaction evaluation of students with the built environment. In contrast, surrounding green vegetation, the low number of semi-open spaces, and the poor view of the semi-open space had no significant effect on the overall satisfaction evaluation.

### 5.2. Natural Exposure and Tendency to Anxiety

At the scale of the academic building, nature contact includes four aspects. These are the surrounding landscape and leisure facilities, surrounding green vegetation, the number of semi-open spaces, and landscape views of semi-open spaces, reflecting different levels of contact with nature [64]: observing nature, being with nature, and seeking physical interaction with nature. However, only the view of the semi-open space landscape significantly affects the probability of generating students’ anxiety tendencies.

Consistent with previous research, this study found that students who were satisfied with the view of the semi-open space were less likely to develop anxiety. Observing nature reduces stress via psychophysiological pathways and is beneficial to mental health [66], and there is a positive correlation between natural window views and improved mental health [67,68,69,70]. Semi-open spaces can also provide multisensory experiences, such as landscape views, bird sounds, and flower fragrances [71], which can improve mental health through many senses [72]. Semi-open spaces allow people to interact with nature during an epidemic, verifying and supplementing existing research on the advantages for mental health of exposure to nature [32].

In contrast to the proposed hypothesis, the surrounding landscape, leisure facilities, and green vegetation did not significantly affect the students’ tendency to be anxious. That differs from previous research, which found that plants provide psychological recovery benefits [73] and that engagement with nature is also beneficial for psychological well-being [74,75]. That could be due to the outbreak’s particular situation and the issue of natural exposure accessibility. In the context of the epidemic, concentrated outdoor activities for students were discouraged, and the leisure facilities of the surrounding landscape and green vegetation had relatively poor accessibility for students in academic buildings, raising the expense of students’ exposure to nature. Therefore, regardless of the students’ satisfaction with the leisure facilities in the surrounding landscape and green vegetation, their tendency to be anxious was unaffected. For pupils studying in the classroom, on the other hand, the semi-open space had better accessibility and convenience and offered a natural shelter from wind and rainy weather. In a context where public activities are discouraged, the natural contact afforded by semi-open spaces is critical for students’ emotional regulation. On the other hand, maintaining social distance measures in the context of the epidemic is an option for the government to help reduce transmission. A lack of socialization may harm students’ mental health [76,77]. Semi-open spaces provide a certain level of social interaction [22], which benefits mental health.

For students’ mental health in the context of the epidemic, built and environmental design and renovation based on mental health should pay more attention to the accessibility and convenience of natural contact facilities, reach a certain amount, have a good landscape view and thermal environment, and provide a certain amount of space for social interaction. Additionally, the outdoor landscape and recreational facilities should be optimized to provide students with total satisfaction with the built environment.

### 5.3. Physical Conditions and Tendency to Anxiety

In terms of physical conditions, only the classroom noise environment and the hot semi-open summer space significantly affected students’ anxiety. In contrast to the previous section’s hypothesis, “classroom ventilation”, “classroom lighting”, “classroom thermal environment”, “surrounding thermal environment”, “surrounding wind environment”, “high wind speed in semi-open space”, and “semi-open space cold in winter”, did not affect students’ anxiety. Additionally, “classroom ventilation”, “classroom thermal environment”, “classroom noise environment”, and “surrounding thermal environment” can significantly affect students’ overall satisfaction ratings of the built environment.

From the perspective of the wind environment, the satisfaction ratings of three aspects, the classroom ventilation, the surrounding wind environment, and high wind speed in semi-open space, had no significant influence on the students’ tendency to anxiety. That is different from previous research, which shows that inadequate ventilation results in air pollution and lowers indoor air quality, which can impact mental health [20]. Air pollution has been linked to poor mental health [41,42,43] as well as behavioral determinants of mental health [51,52], and PM2.5 in air pollution has been associated with anxiety and depression symptoms [43]. The difference in the results of this article could be due to students’ selective behavior to improve ventilation or avoid being in an unfavorable wind environment when they are unsatisfied with the wind environment. Students can improve ventilation by employing mechanical exhausts or opening windows, and in response to poor external wind conditions, students may choose to remain indoors. Studies have shown that poor ventilation can impact mental health. However, students are also less likely to suffer from anxiety when ventilation can be controlled and improved, or when avoidance is an option.

From the thermal environment perspective, students who perceived “semi-open space hot in summer” as a factor of dissatisfaction were more likely to develop anxiety tendencies. That is consistent with existing research that suggests summer heat waves may trigger anxiety due to emotional and physical discomfort [78]. Additionally, studies have shown that heat exposure harms mental health, with elevated body temperature linked to depression and anxiety [54]. However, students’ satisfaction ratings of “classroom thermal environment”, “surrounding thermal environment”, and “semi-open space cold in winter” do not affect their anxiety tendencies. It could be because students can improve the thermal environment indoors by using air conditioning and ventilation or choose to be inside when thermal comfort is poor. They can regulate by adding clothes in semi-open spaces where it is cold in the winter. As a result, students’ satisfaction with the classroom thermal environment and surrounding thermal environment, as well as their attitude toward “semi-open space cold in winter”, do not affect their anxiety. On the other hand, “semi-open spaces hot in summer” is more challenging to regulate artificially. The semi-open spaces are convenient and necessary natural contact spaces for students in the context of the epidemic. Thus, students who are dissatisfied with the heat in the semi-open spaces in the summer are more likely to develop anxiety.

From the perspective of the noise environment, students’ satisfaction ratings of the classroom noise environment were found to have a significant inverse effect on their anxiety tendencies. That is consistent with current research findings corroborating the correlation of noise with depression and anxiety [79] and the negative impact on mental health [59,60]. When students are dissatisfied with the noisy environment but unable to change or control it, they develop anxiety. Students’ satisfaction with the noisy environment is lowest on university campuses in cities, which are typically more bothered by city and traffic noise. Because urban and traffic noise is linked to poor mental health outcomes [30], students who are dissatisfied with their noisy surroundings are more likely to feel anxious. The study’s findings also reveal flaws in the present campus’s noise prevention plan. Treatment for noise prevention should be considered throughout planning, building design, and material construction.

From the perspective of the lighting environment, students’ satisfaction ratings of the classroom lighting had no significant effect on the development of anxiety tendencies, and most students were satisfied with classroom lighting. Even with poor lighting, students can control and improve it by turning on the lights or pulling the curtains. Although increasing exposure to natural light has antidepressant effects [80] and human vision is usually better in daylight than in electric lighting [61], non-sunlit areas do not always cause visual discomfort [63]. As a result, students dissatisfied with the illumination are less likely to be anxious.

This paper’s findings on the relationship between students’ satisfaction ratings of physical conditions and anxiety tendencies corroborate previous studies. Mental health outcomes are related to an individual’s ability to control their physical self and surroundings [81,82,83,84]. Control over the built environment can alter an individual’s mental health through direct pathways [20]. Thus, whether student satisfaction ratings of physical conditions affect anxiety tendencies may depends on whether improvements in physical conditions are controlled or can be avoided without loss. That explains why unsatisfactory ratings of the lighting, wind conditions and thermal environments do not produce student anxiety. However, “classroom noise” and “summer heat in semi-open spaces” are difficult to improve or avoid, so students’ dissatisfaction can easily lead to anxiety. As a result, there is an urgent need for targeted design and modification of classroom noise and summer heat protection in semi-open areas to improve students’ mental health and minimize anxiety. Moreover, students’ ratings of classroom ventilation, the thermal environment, the noise environment, and the surrounding thermal environment all significantly impact their overall satisfaction with the built environment. Consequently, there is a need to improve them in the design and renovation of the built environment.

### 5.4. General Satisfaction with the Built Environment and Anxiety Tendencies

After controlling for the confounding effects of other built environment factors, general satisfaction with the built environment can negatively and significantly influence students’ anxiety tendencies, confirming previous research on the crucial role of the built environment on mental health (e.g., anxiety and depression) [8,9,10,11]. It also shows that, in addition to the built environment factors summarized in this study, the built environment, as a physical environment created for human life and activity, contains other factors that may influence students’ tendency to anxiety. For example, preferred interior colors, indoor plants, and artwork with windows can reduce the risk of anxiety and depression [28]. On the other hand, students’ anxiety has an inverse effect on overall satisfaction evaluation of the built environment. However, the magnitude of the effect is small, indicating that students’ anxiety can have some influence on the evaluation and judgment of satisfaction.

### 5.5. Limitations and Future Work

This study has certain limitations. We collected the data through an online questionnaire. Students were exposed to various irrelevant elements when filling out the questionnaire, which may have resulted in a discrepancy with the students’ actual psychological state. As mentioned above, other factors of the built environment deserve further research, such as interior layout, color, vignettes, different decorative settings, cultural shaping, and behavioral thinking, that could be included in future research efforts as variables affecting mental health. Additionally, with the normalization of epidemic prevention, whether there is a change in the satisfaction rating of academic buildings on the impact of student anxiety, further research based on this study can be conducted in the future to accurately analyze the impact of the built environment on mental health and provide a reference for a healthier campus design.

## 6. Conclusions

In this study, the built environment is used as a research perspective at the scale of academic buildings. Students’ satisfaction ratings of various features of the built environment of university teaching buildings in the context of the epidemic and the impact of these satisfaction ratings on students’ anxiety tendencies were counted and analyzed using online questionnaire data. The built environment factors studied included three dimensions of the academic building’s indoor, semi-open space, and outdoor perimeter, including both natural contact and physical conditions, as well as demographic background variables, for a total of 16 variables included in the final multiple linear regression and binary logistic regression models, respectively. Additionally, the study developed a multi-factor, holistic model to avoid the interference of confounding factors.

From the perspective of nature exposure, the results of this study showed that students who were dissatisfied with the poor view of semi-open spaces were more at risk of anxiety. At the same time, satisfaction ratings of the surrounding landscape and leisure facilities, the surrounding green vegetation, and the low number of semi-open spaces had no significant effect on students’ anxiety tendencies. From the viewpoint of physical conditions, the satisfaction rating of the classroom noise environment significantly and negatively impacted students’ anxiety tendencies. Students dissatisfied with the hot semi-open space in summer were more at risk of anxiety. In contrast, the satisfaction rating of the classroom ventilation, lighting, thermal environment, and the surrounding thermal environment had no significant impact on students’ anxiety tendencies. In addition, the evaluation of the general satisfaction with the built environment also significantly negatively impacted students’ anxiety.

This study also found that the following factors significantly influenced students’ general satisfaction with the academic building’s built environment: classroom ventilation, noise environment, thermal environment, surrounding thermal environment, and the landscape and leisure facilities.

In the context of the epidemic, the architectural design and renovation of academic buildings based on mental health should prioritize the design of semi-open spaces. Designs should have a good view of the landscape and a better thermal environment in summer, in addition to improving the design of classrooms against noise. Additionally, improving classroom ventilation, the thermal environment, the noise environment, the surrounding thermal environment, and surrounding landscape facilities are all essential to enhance students’ overall satisfaction with the built environment.

Students often suffer from unfavorable psychological emotions in the context of the epidemic and the campus’s local management. The study’s findings can guide the architectural design and renovation of related academic buildings to increase students’ satisfaction with the built environment, improve psychological health, and alleviate anxiety.

## Figures and Tables

**Table 1:** Built environment factors.

Three Dimensions	Built Environment Subfactor	Two Aspects
Indoor classroom environment in the academic building	Classroom ventilation	Physical Conditions
	Classroom lighting	Physical Conditions
	Classroom thermal environment	Physical Conditions
	Classroom noise environment	Physical Conditions
Semi-open space environment of the academic building	Number of semi-open spaces	Natural Exposure
	Semi-open space landscape view	Natural Exposure
	Semi-open space wind environment	Physical Conditions
	Semi-open space thermal environment	Physical Conditions
Outdoor environment around the academic building	Surrounding landscape and leisure facilities	Natural Exposure
	Surrounding green vegetation	Natural Exposure
	Surrounding wind environment	Physical Conditions
	Surrounding thermal environment	Physical Conditions

**Table 2:** Variables and questionnaires.

Category	Variables	Question	Variable Assignment
Demographic background variables	Gender	Q1_1 What is your gender?	Male = 1, Female = 2
	Grade	Q1_2 What grade are you in?	First year = 1, second year = 2, third year = 3, fourth year = 4, fifth year = 5, graduate = 6
	Climate zone	Q1_3 What is your school?	Open questions
	Inner or outer corridor	Q1_4 Is the academic building you evaluated an inner corridor or an outer corridor?	Outer corridor = 0, inner corridor = 1
Dependent variable	Anxiety tendencies	The Generalized Anxiety Disorder scale (GAD-7)	Without anxiety tendencies (GAD-7 score < 5) = 0, with anxiety tendencies (GAD-7 score ≥ 5) = 1
	General satisfaction	Q2_2 Are you generally satisfied with the completed environment of the academic building?	Very dissatisfied = 1, not very satisfied = 2, average = 3, more satisfied = 4, very satisfied = 5
Indoor built environment of classrooms in academic buildings	Classroom ventilation	Q3_1 Are you satisfied with the ventilation of the classroom?	Very dissatisfied = 1, not very satisfied = 2, average = 3, more satisfied = 4, very satisfied = 5
	Classroom lighting	Q3_2 Are you satisfied with the lighting of the classroom?	Very dissatisfied = 1, not very satisfied = 2, average = 3, more satisfied = 4, very satisfied = 5
	Classroom thermal environment	Q3_3 Are you satisfied with the hot and cold comfort of the classroom?	Very dissatisfied = 1, not very satisfied = 2, average = 3, more satisfied = 4, very satisfied = 5
	Classroom noise environment	Q3_4 Are you satisfied with the classroom noise environment?	Very dissatisfied = 1, not very satisfied = 2, average = 3, more satisfied = 4, very satisfied = 5
Outdoor built environment around the academic building	Surrounding landscape and leisure facilities	Q4_1 Are you satisfied with the landscape and recreational facilities around the academic building?	Very dissatisfied = 1, not very satisfied = 2, average = 3, more satisfied = 4, very satisfied = 5
	Surrounding green vegetation	Q4_2 Are you satisfied with the environment of greenery and vegetation around the academic building?	Very dissatisfied = 1, not very satisfied = 2, average = 3, more satisfied = 4, very satisfied = 5
	Surrounding wind environment	Q4_3 Are you satisfied with the wind environment around the academic building?	Very dissatisfied = 1, not very satisfied = 2, average = 3, more satisfied = 4, very satisfied = 5
	Surrounding thermal environment	Q4_4 Are you satisfied with the hot and cold environment around the academic building?	Very dissatisfied = 1, not very satisfied = 2, average = 3, more satisfied = 4, very satisfied = 5
Built environment of semi-open space of academic building	Semi-open space satisfaction	Q5_1 Are you satisfied with the semi-open space of the academic building?	Very dissatisfied = 1, not very satisfied = 2, average = 3, more satisfied = 4, very satisfied = 5
	Low number of semi-open spaces	Q5_2 Do you think the low number of semi-open spaces is an unsatisfactory factor?	No = 0, yes = 1
	High wind speed in semi-open space	Q5_3 Do you think the high wind speed in semi-open space is an unsatisfactory factor?	No = 0, yes = 1
	Poor view of semi-open space	Q5_4 Do you think the poor view of semi-open space landscape is an unsatisfactory factor?	No = 0, yes = 1
	Semi-open space cold in winter	Q5_5 Do you think the semi-open space cold in winter is an unsatisfactory factor?	No = 0, yes = 1
	Semi-open space hot in summer	Q5_6 Do you think semi-open space heat in summer is an unsatisfactory factor?	No = 0, yes = 1

**Table 3:** Descriptive statistics of demographic variables and background characteristics.

		Number of Samples (%)	Number of People with Anxiety (%)
Total		241 (100.0%)	54 (22.4%)
Gender	Male student	110 (45.6%)	25 (46.3%)
	Female student	131 (54.4%)	29 (53.7%)
Grade	First year	14 (5.8%)	1 (1.9%)
	Second year	17 (7.1%)	2 (3.7%)
	Third year	51 (21.2%)	15 (27.8%)
	Fourth year	108 (44.8%)	22 (40.7%)
	Fifth year	17 (7.1%)	5 (9.3%)
	Graduate student	34 (14.1%)	9 (16.7%)
Climate zone	Severe cold regions	7 (2.9%)	3 (5.6%)
	Cold regions	29 (12.0%)	7 (13.0%)
	Hot summer and cold winter regions	139 (57.7%)	24 (44.4%)
	Hot summer and warm winter regions	66 (27.4%)	20 (37.0%)
Inner or outer corridor	Inner corridor	187 (77.6%)	43 (79.6%)
	Outer corridor	54 (22.4%)	11 (20.4%)

**Table 4:** Description of built environment factor satisfaction.

	Average Value	Variables	Average Value	Standard Deviation
Total satisfaction	3.49	General satisfaction	3.49	0.913
Indoor classroom environment in the academic building	3.425	Classroom ventilation	3.42	0.959
		Classroom lighting	3.62	0.896
		Classroom thermal environment	3.37	1.021
		Classroom noise environment	3.29	0.996
Outdoor environment around the academic building	3.52	Surrounding landscape and leisure facilities	3.33	1.040
		Surrounding green vegetation	3.73	0.929
		Surrounding wind environment	3.61	0.888
		Surrounding thermal environment	3.41	0.886
Semi-open space environment	3.39	Semi-open space satisfaction	3.39	0.964

**Table 5:** Collinearity diagnostics.

	Independent Variable	Co-Linear Statistics	
		Tolerances	VIF
Q1_1	Gender	0.893	1.120
Q1_2	Grade	0.868	1.153
Q1_3	Climate Zone	0.824	1.214
Q1_4	Inner or outer corridor	0.958	1.044
Q2_2	General satisfaction	0.395	2.529
Q3_1	Classroom ventilation	0.391	2.554
Q3_2	Classroom lighting	0.376	2.660
Q3_3	Classroom thermal environment	0.399	2.504
Q3_4	Classroom noise environment	0.481	2.078
Q4_1	Surrounding landscape and leisure facilities	0.360	2.778
Q4_2	Surrounding green vegetation	0.304	3.289
Q4_3	Surrounding wind environment	0.270	3.702
Q4_4	Surrounding thermal environment	0.330	3.026
Q5_1	Semi-open space satisfaction	0.379	2.641
Q5_2	Low number of semi-open spaces	0.746	1.341
Q5_3	High wind speed in semi-open space	0.808	1.238
Q5_4	Poor view of semi-open space	0.827	1.209
Q5_5	Semi-open space cold in winter	0.567	1.765
Q5_6	Semi-open space hot in summer	0.552	1.810

**Table 6:** Correlation matrix.

		Q2_2	Q3_1	Q3_2	Q3_3	Q3_4	Q4_1	Q4_2	Q4_3	Q4_4	Q5_1
Q2_2	General satisfaction	1									
Q3_1	Classroom ventilation	0.616 **	1								
Q3_2	Classroom lighting	0.560 **	0.681 **	1							
Q3_3	Classroom thermal environment	0.594 **	0.588 **	0.625 **	1						
Q3_4	Classroom noise environment	0.578 **	0.577 **	0.584 **	0.596 **	1					
Q4_1	Surrounding landscape and leisure facilities	0.618 **	0.537 **	0.494 **	0.428 **	0.485 **	1				
Q4_2	Surrounding green vegetation	0.557 **	0.495 **	0.543 **	0.448 **	0.490 **	0.722 **	1			
Q4_3	Surrounding wind environment	0.532 **	0.470 **	0.537 **	0.490 **	0.473 **	0.677 **	0.765 **	1		
Q4_4	Surrounding thermal environment	0.608 **	0.559 **	0.557 **	0.652 **	0.555 **	0.546 **	0.575 **	0.692 **	1	
Q5_1	Semi-open space satisfaction	0.594 **	0.437 **	0.489 **	0.450 **	0.480 **	0.632 **	0.650 **	0.671 **	0.612 **	1

**: Significant correlation at the 0.01 level (two-tailed).

**Table 7:** Chi-square test with anxiety tendencies.

Categorical Independent Variables		Pearson’s Cardinal Values	Progressive Significance (Bilateral)
Q1_1	Gender	0.012	0.913
Q1_2	Grade	5.483	0.360
Q1_3	Climate Zone	6.213	0.102
Q1_4	Inner or outer corridor	0.166	0.684
Q5_2	Low number of semi-open spaces	9.378	0.002
Q5_3	High wind speed in semi-open space	0.033	0.855
Q5_4	Poor view of semi-open space	14.260	0.000
Q5_5	Semi-open space cold in winter	0.030	0.863
Q5_6	Semi-open space hot in summer	5.019	0.025

**Table 8:** Independent sample test with anxiety tendencies.

		Equivalence of Means *t*-Test	
		*t*	Sig. (Twin-Tailed)
Q2_2	General satisfaction	3.722	0.000
Q3_1	Classroom ventilation	2.048	0.042
Q3_2	Classroom lighting	1.798	0.073
Q3_3	Classroom thermal environment	2.802	0.006
Q3_4	Classroom noise environment	3.643	0.000
Q4_1	Surrounding landscape and leisure facilities	2.543	0.012
Q4_2	Surrounding green vegetation	1.612	0.108
Q4_3	Surrounding wind environment	1.073	0.284
Q4_4	Surrounding thermal environment	2.904	0.004
Q5_1	Semi-open space satisfaction	1.907	0.058

**Table 9:** Chi-square test with general satisfaction.

Categorical Independent Variables		Pearson’s Cardinal Values	Progressive Significance (Bilateral)
Q1_1	Gender	3.149	0.533
Q1_2	Grade	14.924	0.781
Q1_3	Climate zone	25.846	0.011
Q1_4	Inner or outer corridor	0.166	0.684
Q2_1	Anxiety tendencies	17.245	0.002
Q5_2	Low number of semi-open spaces	20.134	0.000
Q5_3	High wind speed in semi-open space	1.579	0.813
Q5_4	Poor view of semi-open space	13.198	0.01
Q5_5	Semi-open space cold in winter	5.198	0.268
Q5_6	Semi-open space hot in summer	6.878	0.142

**Table 10:** Model Table.

	Linear Regression		Binary Logistic Regression	
Dependent Variable	General Satisfaction		Anxiety Tendencies	
Category	Independent Variable		Independent Variable	
Demographic background variables	Q1_1	Gender	Q1_1	Gender
	Q1_2	Grade	Q1_2	Grade
	Q1_3	Climate zone	Q1_3	Climate zone
	Q1_4	Inner or outer corridor	Q1_4	Inner or outer corridor
Anxiety, total satisfaction	Q2_1	Anxiety tendencies	Q2_2	General satisfaction
Indoor classroom environment in academic buildings	Q3_1	Classroom ventilation	Q3_1	Classroom ventilation
	Q3_2	Classroom lighting	Q3_2	Classroom lighting
	Q3_3	Classroom thermal environment	Q3_3	Classroom thermal environment
	Q3_4	Classroom noise environment	Q3_4	Classroom noise environment
Outdoor environment around the academic building	Q4_1	Surrounding landscape and leisure facilities	Q4_1	Surrounding landscape and leisure facilities
	Q4_2	Surrounding green vegetation	Q4_2	Surrounding green vegetation
	Q4_4	Surrounding thermal environment	Q4_4	Surrounding thermal environment
Semi-open space environment of academic building	Q5_1	Semi-open space satisfaction	Q5_1	Semi-open space satisfaction
	Q5_2	Low number of semi-open spaces	Q5_2	Low number of semi-open spaces
	Q5_4	Poor view of semi-open space	Q5_4	Poor view of semi-open space
	Q5_6	Semi-open space hot in summer	Q5_6	Semi-open space hot in summer

**Table 11:** Coefficients of multiple linear regression model.

	Unstandardized coefficient		Standardization Factor	*t*	Significance	Covariance Statistics	
	B	Standard Error	Beta			Tolerances	VIF
(Constant)	0.536	0.174		3.084	0.002		
Q4_1 Surrounding landscape and leisure facilities	0.255	0.048	0.290	5.307	0.000	0.608	1.645
Q3_3 Classroom thermal environment	0.157	0.056	0.176	2.810	0.005	0.464	2.155
Q3_1 Classroom ventilation	0.185	0.057	0.194	3.252	0.001	0.510	1.961
Q4_4 Surrounding thermal environment	0.154	0.064	0.150	2.397	0.017	0.466	2.145
Q3_4 Classroom noise environment	0.127	0.054	0.138	2.365	0.019	0.532	1.881

**Table 12:** Binary logistic regression model.

Omnibus tests of model coefficients	Cardinality	63.66
	Significance	0.00
Hosmer-Lemeshow test	Cardinality	10.28
	Significance	0.17
−2 Log-Likelihood		184.14
Percentage of correct predictions	No tendency to anxiety	93.10
	Tendency to anxiety	31.40
	Overall percentage	79.90

**Table 13:** Table of coefficients of binary logistic regression model.

Category	Variable Name		B	Standard Error	Wald	Significance	Exp(B)	95% Confidence Interval of EXP(B)	
								Lower Limit	Upper Limit
Demographic background variables	Q1_1	Gender	0.126	0.361	0.121	0.728	1.134	0.559	2.301
	Q1_2	Grade	0.261	0.152	2.961	0.085	1.298	0.964	1.747
	Q1_3	Climate zone	0.313	0.260	1.446	0.229	1.367	0.821	2.275
	Q1_4	Inner or outer corridor	−0.211	0.442	0.228	0.633	0.810	0.341	1.925
Total satisfaction	Q2_2	General satisfaction	−0.559	0.282	30.930	0.047	0.572	0.329	0.994
Indoor classroom environment in academic buildings	Q3_1	Classroom ventilation	0.305	0.272	10.260	0.262	1.356	0.797	2.310
	Q3_2	Classroom lighting	−0.092	0.291	0.100	0.752	0.912	0.516	1.613
	Q3_3	Classroom thermal environment	0.071	0.248	0.081	0.775	1.073	0.661	1.743
	Q3_4	Classroom noise environment	−0.485	0.233	4.317	0.038	0.616	0.390	0.973
Semi-open space environment of academic building	Q4_1	Surrounding landscape and leisure facilities	−0.184	0.263	0.489	0.484	0.832	0.497	1.393
	Q4_2	Surrounding green vegetation	0.322	0.278	1.343	0.247	1.380	0.800	2.380
	Q4_4	Surrounding thermal environment	−0.204	0.305	0.447	0.504	0.816	0.449	1.482
Outdoor environment around the academic building	Q5_1	Semi-open space satisfaction	0.404	0.272	2.205	0.138	1.498	0.879	2.553
	Q5_2	Low number of semi-open spaces	0.718	0.386	3.466	0.063	2.051	0.963	4.370
	Q5_4	Poor view of semi-open space	1.169	0.401	8.507	0.004	3.220	1.468	7.066
	Q5_6	Semi-open space hot in summer	0.867	0.401	4.667	0.031	2.380	1.084	5.228
Constants			−2.845	1.628	3.052	0.081	0.058		

## Data Availability

The datasets generated or analyzed during this study are available from the corresponding author on reasonable request.
